# Motion of Quantum Particles in Terms of Probabilities of Paths

**DOI:** 10.3390/e27070728

**Published:** 2025-07-06

**Authors:** Emilio Santos

**Affiliations:** Departamento de Física, Universidad de Cantabria, 39005 Santander, Spain; santose@unican.es

**Keywords:** quantum particles, path integrals, wave-particle duality, Born approximation, models of quantum physics, Huygens principle, random motion

## Abstract

The Feynman path integral formalism for non-relativistic quantum mechanics is revisited. A comparison is made with cases of light propagation (Huygens’ principle) and Brownian motion. The difficulties for a physical model applying Feynman’s formalism are pointed out. A reformulation is proposed, where the transition probability of a particle from one space-time point to another one is the sum of probabilities of the possible paths. As an application, Born approximation for scattering is derived within the formalism, which suggests an interpretation involving the stochastic motion of a particle rather than the square of a wavelike amplitude.

## 1. Path Integral Formulation of Quantum Mechanics

The path integral formulation of quantum mechanics was introduced by Feynman in 1948 [1]. In a book on the subject [2], the formalism is shown to be a straightforward consequence of the superposition principle. In fact, let us assume that there is a source of particles at point $x=x_{0},\;y=0$, as well as a screen with two slits at x=±a, y=b, and the particles may be detected at any point in the plane y=c. (For simplicity, I ignore here the third coordinate, *z*). If a particle leaves the source at time t=0, crosses a slit at $t_{1}$, and arrives at the detector at $t_{2}$, the probability, P(x), of reaching a point with coordinate *x* in the screen y=c is proportional to the square modulus of a “probability amplitude”, and the latter is the sum of two amplitudes:

(1) $$\begin{array}{ccc} P(x) & \propto & {\left|A(x_{0},0|x,t_{2})\right|}^{2},A\left(x_{0},0|x,t_{2}\right)=\\ & = & A\left(x_{0},0|a,t_{1}\right)A\left(a,t_{1}|x,t_{2}\right)+A\left(x_{0},0|-a,t_{1}\right)A\left(-a,t_{1}|x,t_{2}\right). \end{array}$$

Now, we consider the case of having many screens at positions $y_{1},y_{2},\ldots$, where the particle may arrive at times $t_{1},t_{2},\ldots$, respectively, and every screen possesses many slits, at positions $x_{1},x_{2}$,…In this case the amplitude Equation (1) is replaced by

(2) $$A\left(x_{0},0|x,t\right)=\sum_{j}\sum_{k}\ldots \sum_{q}A\left(x_{0},0|x_{j},t_{1}\right)A\left(x_{j},t_{1}|x_{k},t_{2}\right)\ldots A\left(x_{q},t_{n}|x,t\right).$$

Here, the set of positions $\left\{x_{0},x_{j},x_{k},\ldots x_{q},x\right\}$ may be called a path, meaning that the amplitude $A(x_{0},0|x,t)$ is a sum of amplitudes, every one corresponding to one possible path. If the number of slits in every screen increases indefinitely, at the end, there will be no screen at all. Then, the discrete values $x_{l},x_{k},\ldots$ become continuous, and the sums become integrals, that is

(3) $$A\left(x_{0},0|x,t\right)=\int dx_{1}\cdots \int dx_{n-1}A\left(x_{0},0|x_{1},t_{1}\right)\ldots A\left(x_{n-1},t_{n-1}|x,t\right).$$

The time intervals chosen may be identical, that is $t_{j+1}-t_{j}=\varepsilon ,$ with ε being as small as desired. In the limit ε→0,$A(x_{0},0|x,t)$ becomes an integral of path amplitudes.

The derivation shows that Equation (3) is not specific to Feynman’s formulation of quantum mechanics, but it may be valid for any field fulfilling a superposition property (e.g., classical optics). In this case, however, P(x) of Equation (1) would not be a probability but a light intensity, as will be illustrated below with Huygens’ principle. Also, an expression formally similar to Equation (2) may be used in the study of random motion. Indeed, the probability that a particle goes from one position to another one in some time interval is the sum of probabilities corresponding to different paths. This provides a method for calculations that was pioneered by Norbert Wiener in the study of Brownian motion. This will be illustrated below with a derivation of the diffusion law via a path integral. In this case, the probability density is given directly rather than via the modulus squared of an amplitude. More generally, a path integral formulation might be associated with the evolution of any physical system governed by a linear partial differential equation of first order in the time variable.

What is specific to Feynman’s formulation of (non-relativistic) quantum mechanics is the choice of *A* to be the exponential of i/ħ times the (classical) Lagrangian, *L*, of the particle’s motion, an idea taken from Dirac [3]. For instance in the case of one-dimensional motion in a potential V(x), we have $L=\frac{1}{2}m{\overset{\cdot }{x}}^{2}-V\left(x\right)$

(4) $$\begin{array}{ccc} A\left(x_{j-1},t_{j-1}|x_{j},t_{j}\right) & = & \sqrt{\frac{m}{2\pi i\hbar \varepsilon }}\exp\left(\frac{i\varepsilon }{\hbar }L_{j}\right),\\ L_{j} & \equiv & \frac{1}{2}m{\left(\frac{x_{j}-x_{j-1}}{\varepsilon }\right)}^{2}-\frac{1}{2}\left[V\left(x_{j-1}\right)+V\left(x_{j}\right)\right] \end{array}$$

where *m* is the mass of the particle. This expression differs from the original one of Feynman [2] because I have substituted $\frac{1}{2}\left[V\left(x_{j-1}\right)+V\left(x_{j}\right)\right]$ for $V\left[\left(x_{j-1}+x_{j}\right)/2\right]$ for later convenience. Both formulations agree in the limit ε→0). I shall ignore the terms $V\left(x_{0}\right)$ and $V\left(x\right),$ so that the potential enters n−1 times in Equation (3), as it should. It is common to take the continuous limit and write Equation (2) in the following form:

(5) $$\begin{array}{ccc} A(x_{0},0 & | & x,t)=\int D\left(paths\right)\exp\left[\frac{i}{\hbar }\int_{0}^{t}dtL\left(x,\overset{\cdot }{x}\right)\right]\\ & = & \int D\left(paths\right)\exp\left[\frac{i}{\hbar }\int_{0}^{t}dt\left(\frac{1}{2}m{\overset{\cdot }{x}}^{2}-V\left(x\right)\right)\right]. \end{array}$$

This symbolic equation actually represents the limit ε→0 of Equation (3), with *A* given by Equation (4). The amplitude Equation (5) may be calculated explicitly for simple potentials [2].

The amplitude $A(x_{0},0|x,t)$ is named the “propagator” of the wavefunction. It allows us to obtain the wavefunction at time *t* from the wavefunction at time 0, that is [2],

(6) $$\psi \left(x,t\right)=\int dx_{0}\psi \left(x_{0},t\right)A\left(x_{0},0|x,t\right).$$

Hence, it follows that the propagator fulfils the Schrödinger equation with the initial condition

$$A\left(x_{0},0|x,0\right)=\delta \left(x-x_{0}\right),$$

where $\delta \left(x\right)$ is Dirac’s delta. Thus, the propagator is the Green’s function of the Schrödinger equation.

As is well known, the path integral formulation may be generalized to three dimensions, to many-particle systems, and to relativistic field theory. It has an extremely important role in modern theoretical physics, both because it is well adapted to derive general properties, e.g., symmetries, and due to the relevance for actual calculations, it is the seed of Feynman graphs in covariant perturbation theory [4]. Dealing with formal and calculational aspects lies out of the scope of this article, which is devoted to the physical interpretation of the Feynman formalism in (non-relativistic) quantum mechanics. In particular, below, I will present a possible interpretation of the path integral formalism, but prior to this, I will revisit the use of a formula similar to Equation (3) in classical optics and diffusion theory in order to make a comparison with the Feynman formalism.

## 2. Other Examples of Path Integrals

### 2.1. Huygens’ Principle

Historically the first formulation of a physical theory in terms of “path integrals” goes back to the days of Christiaan Huygens, more than three centuries ago. In fact, Huygens proposed that light consists of waves, in opposition to his contemporary Isaac Newton, who supported a corpuscular theory. He was able to explain the straight line propagation and other properties of light through his celebrated principle. Huygens’ principle states that light propagation may be understood as if every point where light arrives becomes the source of a spherical wave, and the waves coming from different points are able to interfere. In practice, this implies that, from each point of a given wavefront at time *t*, spherical wavelets originate. Thus, Huygens’ principle may be formalized, stating that the light arriving at time *t* at a point r may be calculated from the three-dimensional generalization of Equation (3), with appropriate transition amplitudes $A\left(r_{j},t_{j}|r_{j+1},t_{j+1}\right).$ At odds with the cases of Feynman’s formalism, in Equation (5), or the diffusion equation (see Equation (13) below), where the velocities of the particles may have any value, light travels in vacuum with a fixed velicity, *c*, which leads to the constraint $\left|r_{j+1}-r_{j}\right|=c\left(t_{j+1}-t_{j}\right).$ Hence, the transition amplitude should be of the form

(7) $$A\left(r_{j},t_{j}|r_{j+1},t_{j+1}\right)=f\left(r_{j}|r_{j+1}\right)\delta \left(\left|r_{j+1}-r_{j}\right|-c\left(t_{j+1}-t_{j}\right)\right),$$

where δ() is Dirac’s delta. Light consists of transverse waves (that is, the electric and magnetic fields are perpendicular to the direction of propagation), which implies that the function *f* should depend also on the angle between the electric field and the $r_{j}-r_{j+1}$ vectors. I shall avoid this complication, irrelevant for our purposes, considering longitudinal waves, e.g., sound propagation in air, rather than light.

For monochromatic sound waves in air, the function *f* of Equation (7) is especially simple:

(8) $$f\left(r_{j}|r_{j+1}\right)=\frac{\exp(ik\left|r_{j+1}-r_{j}\right|)}{2\pi i\left|r_{j+1}-r_{j}\right|}.$$

where the denominator takes into account the fact that the intensity from a point source decreases as the inverse of the distance is squared. I stress that, here, the use of complex amplitudes, i.e., the introduction of the imaginary unit number *i*, has no deep meaning; rather, it is just a convenient mathematical procedure to simplify the calculations. Actually, the wave amplitudes might always be represented by real numbers; indeed, the light amplitude may be taken to be the electric field of the radiation, and the amplitude of a sound wave in air may be taken to be the excess pressure.

In practice, it is most common to use very simple “paths” consisting of just three points, namely {$r_{1},r_{2},r$}. For instance, a typical problem solved with that choice is the diffraction by a small hole (see any book of electromagnetic theory, e.g., [5]).

### 2.2. Diffusion

The path integral formulation is most intuitive in the case of diffusion. Let us consider the well-known problem of the random walk. In one dimension, it consists of a particle that travels a distance λ, either towards the left or towards the right with equal probability, with the step taking a time ε. During the next time interval ε, the particle travels a certain distance again, say λ, either to the left or to the right with equal probability, and so on. The problem lies in finding the probability that the particle is at a given distance, say x=lλ, of the origin after *n* steps, with *n* being a natural number and *l* a positive real number, $\left|l\right|$≤n. The problem might be stated by means of a sum of paths probabilities as follows:

(9) $$P\left(x_{0},0|x,t\right)=\sum_{j}\cdots \sum_{q}P\left(x_{0},0|x_{j},\varepsilon \right)P\left(x_{j},\varepsilon |x_{k},2\varepsilon \right)\ldots P\left(x_{q},\left(n-1\right)\varepsilon |x,t\right),$$

where the set of positions $\left\{x_{0},x_{j},x_{k},\ldots x_{q},x\right\}$ represents a path. Remember that $\left|x_{j}-x_{0}\right|=\left|x_{k}-x_{j}\right|=\cdots =\lambda$ and that the probabilies for every step are

(10) $$P\left(x_{s},m\varepsilon |x_{r},\left(m+1\right)\varepsilon \right)=\frac{1}{2}\;\mathrm{if}\;x_{r}-x_{s}=\lambda ,P=\frac{1}{2}\;\mathrm{if}\;x_{r}-x_{s}=-\lambda .$$

The solution may be found as follows. In order to ensure that the particle arrives at a distance lλ from the origin after *n* steps, it is necessary and sufficient that k≡(n+l)/2 steps are towards the right and n−k=(n−l)/2 are towards the left (assuming that the positive direction is to the right.) The set of positions may be called *the path* of the particle. The probability of a given path is ${\left(1/2\right)}^{n}$, and the number of paths leading from 0 to lλ is the combinatorial number nk As a consequence, the probability of reaching lλ in *n* steps is

PmL=nk12n=n!k!n−k!12n=n!(n+l)/2!(n−l)/2!12n.

If *n* and *m* are very large, we may approximate the factorials by the Stirling formula, that is,

$$\log\left(k!\right)≃\frac{1}{2}\log\left(2\pi k\right)+k\log k-k.$$

Ignoring terms which do not depend on *l*, we get

$$\log P_{x}≃const.-\frac{n+l}{2}\log\left(\frac{n+l}{2}\right)-\frac{n-l}{2}\log\left(\frac{n-l}{2}\right).$$

Hence, I obtain

$$P_{x}\propto {\left(n+l\right)}^{\frac{n+l}{2}}{\left(n-l\right)}^{\frac{n-l}{2}}\propto {\left(1-\frac{l^{2}}{n^{2}}\right)}^{n/2}{\left(\frac{n-l}{n+l}\right)}^{m/2}≃\exp\left(-\frac{l^{2}}{2n}\right),$$

where I have taken into account that, in most paths, l<<n for very large *n*. In terms of the total time, t≡nε, and the final position x≡nλ, this may be written as a probability density which, when properly normalized, reads

(11) $$\rho \left(x_{0},0|x,t\right)=\sqrt{\frac{1}{4\pi t}}\exp\left(-\frac{x^{2}}{4Dt}\right),$$

with the parameter $D\equiv \lambda^{2}/\varepsilon$ being the diffusion constant. For us, the relevant conclusion is that the diffusion probability may be calculated as a sum of the probabilities corresponding to all paths leaving the origin at the initial time and arriving at the point *x* at time *t*. The calculation is rather simple because all paths have the same probability.

We may derive the same diffusion probability, starting with a different assumption. That is, in every step of duration ε, the particle may travel any distance, Δx, with a probability proportional to $\exp\left(-\frac{\Delta x^{2}}{2D\varepsilon }\right).$ Then, the probability of reaching *x* will be

(12) $$P\propto \int dx_{1}\int dx_{2}\cdots \int dx_{n-1}\exp\left[-\frac{1}{4D}\sum_{k=0}^{n-1}\frac{{\left(x_{k+1}-x_{k}\right)}^{2}}{t_{m+1}-t_{m}}\right],x_{n}=x,$$

In the limit of large *n*, but fixed t=nε, this may be written by substituting integrals for the sums, that is,

(13) $$P\propto \int D\left(paths\right)\exp\left(\frac{1}{4D}\int_{0}^{t}dt{\overset{\cdot }{x}}^{2}\right),$$

which is formally similar to the Feynman path integral Equation (5). There are however two fundamental differences. Firstly, the diffusion case Equation (13) directly gives the probability, rather than the amplitude, and secondly, the quantity inside the exponential is real and positive, so the sum involves probabilities rather than complex numbers. The integrals in Equation (12) are rather simple, and the result is the probability density Equation (11), which is Green’s function of the diffusion equation

$$\frac{\partial }{\partial t}\rho \left(0,0|x,t\right)=D\frac{\partial^{2}}{\partial x^{2}}\rho \left(0,0|x,t\right).$$

A technique similar to the one illustrated here for diffusion may be applied to any process where the probability of going from an initial state to a final one is the sum of the probabilities of the different paths. This is the case for any random motion, but the fact that the transition probability in a step does not depend on the previous positions is not general. If it is fulfilled, the stochastic process is called Markovian. This property holds true in Equation (9), where the transition probabilities in different steps are statistically independent of each other.

## 3. Physical Interpretation of Feynman’s Path Integrals

It is sometimes stated that Feynman’s path integral formalism provides an intuitive picture of the quantum mechanical evolution. I do not agree. In sharp contrast with the cases of both Huygens’ principle and diffusion, Feynman’s formalism of non-relativistic quantum mechanics is counterintuitive, and this is for two reasons. The first reason is because the paths are sets of disconected points rather than (continuous) paths in the usual sense of the word. The second reason is because, in an intuitive picture, the probability of travel of a particle between two points should involve a sum of probabilities, not amplitudes. I comment on these two shortcomings in the following paragraphs.

The alleged path represented by the positions $\left\{x_{0},x_{1},x_{2}\cdots x_{n},x\right\}$ is actually a set of points, each one separated from the previous one by a distance that is usually very long. Indeed, the position $x_{j-1}$ may have values in the interval $\left(-\infty ,\infty \right),$ which is the interval of integration, with all values having the same weight according to the Riemann measure of the integral (see Equation (3)). Similarly, the position $x_{j}$ may have values in the interval $\left(-\infty ,\infty \right)$, with all values having the same weight. Thus, the step $u_{j}=x_{j}-x_{j-1}$ between two positions may be *arbitrarily large.* Indeed, its mean squared value, $\langle u_{j}^{2}\rangle ,$ diverges. The counterintuitive character is enhanced by the fact that the (indefinitely long) step $u_{j}$ *takes place in an infinitesimal time interval* ε. Thus, Feynman’s Equation (3) should be seen as a purely mathematical construction, a calculational tool where the physical meaning appears only in the final result of the calculation.

In sharp contrast, Huygens’ path integrals, Equation (7), are continuous. In fact, the quantities $\left|r_{j+1}-r_{j}\right|$ are never too large due to the denominator in Equation (8), and above all, they decrease to zero when the time interval, $t_{j+1}-t_{j},$ goes to zero. Thus, the sums or integrals involved are performed over *continuous paths*. A similar thing happens in the diffusion theory defined by Equations (9) and (10). In this case, every path is continuous, although it may not be differentiable. The same is true if we define the path via Equation (12), where the probability that the mean velocity in a step, $\left(x_{k+1}-x_{k}\right)/\left(t_{m+1}-t_{m}\right),$ surpasses a fixed value *K* goes exponentially to zero when K→∞.

From a physical rather than formal point of view, Feynman’s path integral is more similar to Huygens’ principle of classical optics than to the diffusion problem. Indeed, the use of an amplitude suggests a wave picture, although the fact that the modulus squared of the amplitude is a probability, rather than an intensity, gives a particle appearance. In this respect, there is a difficulty for an intuitive picture which is general; indeed, the wave–particle duality is the “main mystery of quantum mechanics”, in Feynman’s words. On the other hand, the interpretation of path integrals for radiation (“photons”) might be considered as an elaboration of Huygens’ principle of classical optics. Then, the main conceptual difference is that, in classical optics, the amplitude square is an intensity (power per unit area), while in Feynman’s formalism, it is assumed to be a probability density (of having a photon). There is also an alternative interpretation assuming that radiation consists of waves and the corpuscle behaviour appears because the probability of detection is proportional to the radiation intensity arriving at the detector [6].

## 4. Transition Probability as a Sum of Paths Probabilities

In the following, I present a formulation for the motion of a quantum particle in terms of probabilities (rather than amplitudes!) of paths, with the condition that the transition probability agrees with the square modulus of the Feynman amplitude Equation (5), that is,

$$P\left(x_{0},0\to x,t\right)={\left|A(x_{0},0|x,t)\right|}^{2}.$$

If we take the (continuous) set of paths as discrete for the sake of clarity, and we generalize to 3 dimensions, our aim is to get the transition probability as a sum of probabilities of paths, that is,

(14) $$P\left(r_{a},t_{a}\to r_{b},t_{b}\right)=\sum_{k}W_{k}\left(r_{a},t_{a}\to r_{b},t_{b}\right).$$

Here, every value of the index *k* corresponds to a possible path of the particle with end points $\left(r_{a},t_{a}\right)$ and $\left(r_{b},t_{b}\right)$. The problem lies in finding “weights” $W_{k}$ which could be interpreted as probabilities in order to provide an intuitive picture of the quantum evolution as a random motion of particles. In the following, I propose a method to get the “weights”. In some cases, to be studied below, the weights are non-negative-definite, and therefore, they may be interpreted as probabilities; in other cases, the formalism should be slightly modified in order to get positivity, as shown at the end of Section 4.1.

I shall start from the 3D generalization of the amplitude Equation (4), that is,

(15) $$\begin{array}{ccc} A\left(x_{a},t_{a}\to x_{b},t_{b}\right) & = & \lim_{\varepsilon \to 0}{\left(\frac{m}{2\pi i\hbar \varepsilon }\right)}^{3n/2}\int dx_{n-1}\cdots \int dx_{1}\\ & & \prod_{j=1}^{n}\exp\left\{\frac{im}{2\hbar \varepsilon }{\left|x_{j}-x_{j-1}\right|}^{2}-\frac{i\varepsilon }{2\hbar }\left[V\left(x_{j-1}\right)+V\left(x_{j}\right)\right]\right\}, \end{array}$$

where $\varepsilon \equiv t_{j}-t_{j-1},$ is independent of *j* and $x_{0}\equiv x_{a},x_{n}\equiv x_{b}.$ The limit ε→0 should be understood with n→∞ fulfilling

(16) $$n\varepsilon =t_{b}-t_{a}.$$

Actually, the integrals involved are not convergent; therefore, an appropriate regularization is implicit.

In quantum mechanics, the transition probability is the square modulus of the transition amplitude, which, in the path integral formalism, becomes

(17) $$\begin{array}{ccc} P(r_{a},t_{a} & \to & r_{b},t_{b})={\left|A(r_{a},t_{a}\to r_{b},t_{b})\right|}^{2}\\ & = & \int D\left(pathpairs\right)\exp\left[\frac{i}{\hbar }\int_{t_{a}}^{t_{b}}dt\left(\frac{1}{2}m{\dot{y}}^{2}-V\left(y\right)\right)\right]\\ & & \times \exp\left[-\frac{i}{\hbar }\int_{t_{a}}^{t_{b}}dt\left(\frac{1}{2}m{\dot{x}}^{2}-V\left(x\right)\right)\right], \end{array}$$

where $x_{0}=y_{0}=r_{a},x_{n}=y_{n}=r_{b},$ and ∫D represents a functional integral. Taking Equation (15) into account, the symbolic equation Equation (17)) may be written explicitly as follows:

(18) $$\begin{array}{ccc} P(r_{a},t_{a} & \to & r_{b},t_{b})=\lim_{\varepsilon \to 0}{\left(\frac{m}{2\pi \hbar \varepsilon }\right)}^{3n}\int dx_{n-1}\cdots \int dx_{1}\int dy_{n-1}\cdots \int dy_{1}\\ & & \times \prod_{j=1}^{n}\exp\left\{\frac{im}{2\varepsilon \hbar }\left[{\left|y_{j}-y_{j-1}\right|}^{2}-{\left|x_{j}-x_{j-1}\right|}^{2}\right]\right\}\\ & & \times \prod_{j=1}^{n}\exp\left\{-\frac{i\varepsilon }{2\hbar }\left[V\left(x_{j}\right)-V\left(y_{j}\right)\right]\right\}, \end{array}$$

where I will represent vectors with bold face letters so that $\int dx_{j}$, $\int dy_{j}$ are triple integrals over the whole 3D space, and I have included the parameters *m* and ħ following Feynman [2]. With that choice, the quantity $P(r_{a},t_{a}\to r_{b},t_{b})$ has dimensions of probability per square volume. Then, the probability that a particle is in some finite volume *B* at time $t_{b}$, conditionally in another finite volume *A* at an earlier time $t_{a}$, will be

$$P\left(A\to B\right)=\int_{r_{a}\in A}dr_{a}\int_{r_{b}\in B}dr_{b}P\left(r_{a},t_{a}\to r_{b},t_{b}\right).$$

In order to proceed, I shall make a change of variables, that is,

(19) $$r_{j}=\frac{1}{2}\left(x_{j}+y_{j}\right),u_{j}=x_{j}-y_{j},\;0\leq j\leq n.$$

Hence, reordering the exponentials, Equation (18) becomes

(20) $$\begin{array}{ccc} P(r_{a},t_{a} & \to & r_{b},t_{b})=\lim_{\varepsilon \to 0}{\left(\frac{m}{2\pi \varepsilon \hbar }\right)}^{3n}\int dr_{n-1}\cdots \int dr_{1}\int du_{n-1}\cdots \int du_{1}\\ & & \times \prod_{j=1}^{n-1}\exp\left[-\frac{im}{\varepsilon \hbar }u_{j}\cdot \left(r_{j-1}-2r_{j}+r_{j+1}\right)\right]\\ & & \times \prod_{j=1}^{n-1}\exp\left\{\frac{i\varepsilon }{2\hbar }\left[V\left(r_{j}-\frac{1}{2}u_{j}\right)-V\left(r_{j}+\frac{1}{2}u_{j}\right)\right]\right\}, \end{array}$$

where $r_{0}=r_{a}$, $r_{n}=r_{b}$, and $u_{0}=u_{n}=0$.

Equation (20) may be written as

(21) $$P\left(r_{a},t_{a}\to r_{b},t_{b}\right)=\lim_{\varepsilon \to 0}{\left(\frac{m}{2\pi \hbar }\right)}^{3}\varepsilon^{-3n}\int dr_{n-1}\cdots \int dr_{1}\prod_{j=1}^{n-1}Q_{j},$$

where

(22) $$\begin{array}{ccc} Q_{j} & \equiv & {\left(\frac{m}{2\pi \hbar }\right)}^{3}\int du\exp\left(-i\frac{m}{\hbar }u\cdot s_{j}\right)\\ & & \times \exp\left\{\frac{i\varepsilon }{2\hbar }\left[V\left(r_{j}-\frac{1}{2}u\right)-V\left(r_{j}+\frac{1}{2}u\right)\right]\right\}, \end{array}$$

with

(23) $$s_{j}\equiv \frac{r_{j-1}-2r_{j}+r_{j+1}}{\varepsilon }=\frac{r_{j+1}-r_{j}}{\varepsilon }-\frac{r_{j}-r_{j-1}}{\varepsilon }\equiv v_{j}-v_{j-1},j=1,2,\ldots n-1.$$

The quantity $s_{j}$ has the physical meaning of velocity change at time *t*, and the ratio $s_{j}/\varepsilon$ might be interpreted as an acceleration. However, the limits ε→0 may not exist; that is, the instantaneous velocity and acceleration are not well defined in general. I will return to this point in Section 4.2 below.

Let us consider several examples. Firstly, I shall study free propagation; when *V* is a constant, Equation (20) becomes

(24) $$\begin{array}{ccc} P\left(a\to b\right) & = & \lim_{\varepsilon \to 0}{\left(\frac{m}{2\pi \varepsilon \hbar }\right)}^{3n}\int dr_{n-1}\cdots \int dr_{1}\int du_{n-1}\cdots \int du_{1}\\ & & \times \prod_{j=1}^{n-1}\exp\left[-\frac{im}{\varepsilon \hbar }u_{j}\cdot \left(r_{j-1}-2r_{j}+r_{j+1}\right)\right], \end{array}$$

where I have taken into account that $u_{0}=u_{n}=0.$ If the $u_{j}$ integrals are applied, we get

(25) $$P\left(a\to b\right)=\lim_{\varepsilon \to 0}{\left(\frac{m}{2\pi \varepsilon \hbar }\right)}^{3}\int dr_{n-1}\cdots \int dr_{1}\prod_{j=1}^{n-1}\delta^{3}\left(r_{j-1}-2r_{j}+r_{j+1}\right).$$

The Dirac deltas $\delta^{3}\left(r_{j-1}-2r_{j}+r_{j+1}\right)$ imply $v_{j}=v_{j-1}$ at all times $t_{j}$, implying a motion with constant velocity. If we perform the integrals in Equation (25), we get

(26) $$P\left(r_{a},t_{a}\to r_{b},t_{b}\right)={\left[\frac{m}{2\pi \hbar \left(t_{b}-t_{a}\right)}\right]}^{3},t=n\varepsilon .$$

In the general case, performing the integrals in $u_{j}$ is not possible without a knowledge of the potential, $V\left(r\right),$ but to the lowest order in ħ, the integrals are simple. In fact, expanding $V\left(r_{j}\pm u_{j}/2\right)$ in powers of $u_{j}$, the exponent in Equation (22) will have

(27) $$V\left(r_{j}-\frac{u_{j}}{2}\right)-V\left(r_{j}+\frac{u_{j}}{2}\right)≃u_{j}.\nabla V\left(r_{j}\right)+O\left(u_{j}^{3}\right),$$

whence integration with respect to u leads to

(28) $$\begin{array}{ccc} P(r_{a},t_{a} & \to & r_{b},t_{b})=\lim_{\varepsilon \to 0}{\left(\frac{m}{2\pi \hbar \varepsilon }\right)}^{3\left(n+1\right)/2}\int dr_{1}\cdots \int dr_{n-1}\\ & & \times \prod_{j=1}^{n-1}\delta^{3}\left(s_{j}+\frac{2\varepsilon }{m}\nabla V\left(r_{j}\right)\right)+O\left(\hbar^{3}\right). \end{array}$$

This corresponds to a motion fulfilling at every time

(29) $$m\frac{r_{j-1}-2r_{j}+r_{j+1}}{\varepsilon^{2}}=-\nabla V\left(r_{j}\right),$$

The above is the (discretized) classical equation of motion. Thus, Equation (28) provides the classical limit of quantum mechanics when ħ→0.

It is interesting that the classical motion, Equation (29), is obtained if the potential, $V(r_{j}),$ is, at most, quadratic in the coordinates because, in this case, Equation (27) is exact (no $O\left(\hbar^{3}\right)$ term appears). This might be interpreted by saying that in “linear problems, the quantum particle follows the classical path”. The typical example is the harmonic oscillator. This is the reason why the quantum mechanics of linear systems look semiclassical. In this case, all quantum effects derive from the fact that the initial wave function cannot be localized in a region that is too small due to the Heisenberg uncertainty principle, a constraint which does not appear in Feynman’s path integral formalism and should be put as an additional constraint. In contrast, it does appear in the standard Hilbert space formalism, where Heisenberg uncertainty is a consequence of the commutation rules.

### 4.1. Path Weights in Terms of the Fourier Transform of the Potential

It is convenient to perform a change leading to a more simple description of the transition probability but equivalent to Equation (20) in the limit ε→0,n→∞. For notational simplicity, in this section, I will remove the dimensional parameters, setting m=ħ=1 and working in one dimension. Restoring the parameters and generalizing to 3D are straightforward processes.

The proposed change consists of starting from

(30) $$\begin{array}{ccc} P & = & \lim_{n\to \infty }{\left(\frac{1}{2\pi \varepsilon }\right)}^{n}\int dz_{1}\cdots \int dz_{n-1}\int du_{1}\cdots \int du_{n-1}\\ & & \times \prod_{j=1}^{n-1}\exp\left[-\frac{i}{\varepsilon }u_{j}\left(z_{j-1}-2z_{j}+z_{j+1}\right)\right]\\ & & \times \prod_{j=1}^{n-1}\exp\left\{\frac{i\varepsilon }{2}\left[V\left(z_{j}-\frac{1}{2}u_{j}\right)-V\left(z_{j}+\frac{1}{2}u_{j}\right)\right]\right\}, \end{array}$$

and getting

(31) $$\begin{array}{ccc} P & = & \lim_{n\to \infty }{\left(\frac{1}{2\pi \varepsilon }\right)}^{n}\int dz_{1}\cdots \int dz_{n-1}\int du_{1}\cdots \int du_{n-1}\\ & & \times \prod_{j=1}^{n-1}\exp\left[-\frac{i}{\varepsilon }u_{j}\left(z_{j-1}-2z_{j}+z_{j+1}\right)\right]\\ & & \times \prod_{j=1}^{n-1}\left\{1+i\frac{i\varepsilon }{2}\left[V\left(z_{j}-\frac{1}{2}u_{j}\right)-V\left(z_{j}+\frac{1}{2}u_{j}\right)\right]\right\}, \end{array}$$

The proof of equivalence follows. Expanding the exponentials of the latter product of Equation (30) in powers of the small parameter ε, we get

(32) $$\exp\left(i\varepsilon B_{j}\right)=1+i\varepsilon B_{j}-\frac{\varepsilon^{2}}{2}B_{j}^{2}-\frac{i\varepsilon^{3}}{6}B_{j}^{3}+\ldots ,$$

where, for brevity, I have labeled

$$B_{j}\equiv \frac{1}{2}\left[V\left(z_{j}-\frac{1}{2}u_{j}\right)-V\left(z_{j}+\frac{1}{2}u_{j}\right)\right].$$

The relevant result is that only the term of order ε in Equation (32) contributes to Equation (30) in the limit ε→0. In fact, we have

(33) $$\prod_{j}\left(1+i\varepsilon B_{j}\right)=1+i\varepsilon \sum_{j}B_{j}-\frac{\varepsilon^{2}}{2}\sum_{j}B_{j}^{2}+\cdots -\varepsilon^{2}\sum_{l}\sum_{j>l}B_{l}B_{j}+\ldots$$

As the sum $\sum_{j}B_{j}$ consists of n−1 terms, the quantity $\varepsilon \sum_{j}B_{j}$ has a finite limit when ε→0 (remember that we assume $n\varepsilon \to t_{b}-t_{a}$). Similarly, there are n(n−1)/2 terms in the double sum $\sum_{l}\sum_{j>l}B_{l}B_{j}$ so that its product times $\varepsilon^{2}$ also has a finite limit. The same happens for those terms on the right side of Equation (33), having as many *different*  $B_{j}$ values as the power of ε. In sharp contrast, the terms containing $B_{j}^{s}$ in Equation (32) with s>1 have extra factors ε, whence they do not contribute in the limit ε→0. For instance, there will be $n\left(n-1\right)$ terms of order $\varepsilon^{3}$ not included in the sum Equation (33), namely those of the form

$$\left(i\varepsilon B_{j}\right)\left(-\frac{\varepsilon^{2}}{2}B_{k}^{2}\right)=-\frac{i\varepsilon^{3}}{2}B_{j}B_{k}^{2}.$$

The sum of those terms contributes a quantity of order $n^{2}\varepsilon^{3}$ that will go to zero in the limit ε→0. A similar argument is valid for s>3. This completes the proof that Equation (30) is equivalent to Equation (31).

The generalization of Equation (31) for three dimensions with the parameters *m* and ħ restored is shown in Equation (21), with $Q_{j}$ now being as follows:

(34) $$\begin{array}{ccc} Q_{j} & \equiv & {\left(\frac{m}{2\pi \hbar }\right)}^{3}\int du\exp\left(-i\frac{m}{\hbar }u\cdot s_{j}\right)\\ & & \times \left\{1+\frac{i\varepsilon }{2\hbar }\left[V\left(r_{j}-\frac{1}{2}u\right)-V\left(r_{j}+\frac{1}{2}u\right)\right]\right\}. \end{array}$$

There are three terms, the first one being a 3D Dirac delta, $\delta^{3}\left(s_{j}\right)$. The integral of u in the second term may be obtained after the change of variables:

$$u=2r_{j}-2x,$$

leading to

$$\begin{array}{ccc} & & {\left(\frac{m}{2\pi \hbar }\right)}^{3}\times \frac{i\varepsilon }{2\hbar }\int du\exp\left(-i\frac{m}{\hbar }u\cdot s_{j}\right)V\left(r_{j}-\frac{1}{2}u\right)\\ & = & \frac{im^{3}\varepsilon }{2\hbar^{4}}\exp\left(-i\frac{2m}{\hbar }r_{j}\cdot s_{j}\right)\int dxV\left(x\right)\exp\left(i\frac{2m}{\hbar }x\cdot s_{j}\right)\\ & = & \frac{im^{3}\varepsilon }{2\hbar^{4}}\exp\left(-i\frac{2m}{\hbar }r_{j}\cdot s_{j}\right)\tilde{V}\left(\frac{2ms_{j}}{\hbar }\right). \end{array}$$

The third term is analogous, and we get the following transition probability

(35) P(ra,ta→rb,tb)=limε→0m2πħ3ε−3n∫drn−1⋯∫dr1∏j=1n−1Qj,Qj=Dj+εFjDj≡δ3sj,Fj≡−m32ħ4ImV˜2msjħexp−2imsj·rjħ,

where $r_{o}=r_{a},r_{n}=r_{b},$ and $s_{j}$ is the change of velocity at time $t_{j};$ see Equation (23). The Fourier transform here is defined as follows:

(36) $$\tilde{V}\left(w\right)\equiv \int dx\exp\left(iw.x\right)V\left(x\right),$$

where w and x are 3D vectors. Calculation of the transition probability $P(r_{a},t_{a}\to r_{b},t_{b})$ is involved because the velocity changes, and the positions are related via Equation (23).

An interesting question is whether the paths involved are continuous. It seems that, here, we have a problem similar to the one in the original Feynman expression for the amplitude, Equations (3) and (4), which I commented on in Section 3. That is, both the integrals of $r_{j}$ and $r_{j+1}$ are extended over the whole space so that the difference $r_{j+1}-r_{j}$ may take any value. It may seem that the same happens in Equation (35) for any pair of consecutive points, whence the path would consist of a set of disjoint points. However, there is a constraint. Firstly, if the quantity $F_{j}$ is nil, then $s_{j}$ is zero with unit probability, whence the path is continuous at that point. Let us examine the case $F_{j}\neq 0.$ If two consecutive points fulfil $r_{j+1}\neq r_{j}$ in the limit ε→0, then $s_{j}$ is divergent; hence, $F_{j}$ is not well defined. I conclude that Equation (35) is sensible only for continuous paths. I conclude that the paths are continuous. The function $r\left(t\right)$ representing a path is both continuous and differentiable at once. The second derivative, which would represent the instantaneous acceleration, is not well defined.

### 4.2. Positivity of the Path Weights

Writing the transition probability in the form of Equation (35) is appropriate in order to study the positivity of the path weights, as shown in the following. If all $F_{j}$ were nil, then the motion of the particle would be uniform, that is, a straight line with constant velocity. However, if $F_{j}\neq 0$, then there is some probability that the motion departs from uniform, with the departure being small at every time $t_{j}$ because $\varepsilon F_{j}$ is small. Furthermore, the contribution $\varepsilon F_{j}$ to the transition probability decreases towards zero when ε diminishes. However, the number *n* of discrete times $\left\{t_{j}\right\}$ increases because $\lim\left(n\varepsilon \right)=t_{b}-t_{b}$ is fixed. Hence, the total contribution of the terms $\left\{F_{j}\right\}$ is finite.

Up to now, the proposed formalism has two shortcomings, one mathematical and the other one physical. The former has to do with the integration of non-convergent integrals, and the latter with the lack of positivity of the path weights. Both may be solved if we regularize the divergent integrals, although this will involve a departure from the Feynman formalism (and thus a deviation from quantum mechanics). In fact, the positivity of the path weights may be guaranteed if we regularize the integrals via substituting $\exp\left(-\gamma m\left|u\right|/\hbar \right)du$ for du in Equation (34), where γ>0 is a small parameter. Then, $D_{j}$ becomes

Dj=m2πħ3∫duexp−imħu·sjexp−γmħu,=m2πħ3Re∫0π2πsinθdθ∫0∞u2duexp−imsjħucosθexp−γmuħ,

where θ is the angle between the vectors u and $s_{j}$. After some algebra, we get

$$D_{j}=\frac{\gamma }{\pi^{2}\left(\gamma^{2}+s_{j}^{2}\right)},$$

which, in the limit γ→0, would become a Dirac delta equivalent to $\delta^{3}\left(s_{j}\right)$ of Equation (35). A similar regularization should be applied to the term $F_{j},$ but it will produce just a small change, and I shall not work with it here. The relevant result is that, for any fixed value of γ>0, there are values of ε small enough so that $\varepsilon \left|F_{j}\right|<D_{j}$, whence $Q_{j}\left(s_{j}\right)>0,$ except maybe for very high values of $s_{j}$. This roughly guarantees the positivity of the path weights in our formalism, at least for smooth and not excessively large potentials $V\left(r\right).$ A more rigorous study of the positivity problem would be worthwhile, but it will not be made in this article.

The normalization of the transition probability equation, Equation (35), derives from the Feynman proposal [2]; see Equation (4). It may not be appropriate for the applications studied in the following sections (Section 4.3, Section 4.4 and Section 4.5). However, I shall not study the problem further; I will get the results with arbitrary normalization.

In summary, the lack of a correct normalization and regularization, Equations (35) and (36), may be interpreted stating that the transition probability is the sum of probabilities of paths. It is interesting that the probability of every path depends on the Fourier transform $\tilde{V}\left(2s_{j}\right)$ of the potential, $V\left(r\right),$ whence the action of $V\left(r\right)$ on the motion of the particle is non-local. A possible explanation for this fact will be put forward in the Discussion and Section 5.

### 4.3. Scattering Experiments

An application of the formalism proposed here is the study of a particle’s scattering by a potential. In those experiments, a source emits particles with velocity $v_{a}$. The particles cross a target region, where they are under the action of a potential $V\left(r\right).$ Then, the particles emerge from the target with different velocities $\left\{v_{b}\right\}$ and eventually arrive at a detector. The target is, in practice, small (microscopic), while the distances from the target to either the source or the detector are both large (macroscopic). In the formalism of this article, I assume that the particles are small (or pointlike) corpuscles. No waves appear.

The quantity of interest in scattering experiments is the differential cross section, $\sigma \left(\theta ,\phi \right)$. It is proportional to the number of particles per unit solid angle, leaving the target with a velocity $v_{b}$ in the direction determined by the angles $\left(\theta ,\phi \right)$. In our formalism, we may write

(37) $$\sigma \left(\theta ,\phi \right)\propto \int dr_{a}\rho \left(r_{a}\right)\int v_{b}^{2}dv_{b}P_{v}\left(r_{a}\to v_{b}\right),$$

where $P_{v}\left(v_{b}\right)$ is the probability that a particle emerging from the point $r_{a}$ of the source with velocity $v_{a}$ reaches the velocity $v_{b}$ after crossing the target. The integral with respect to the modulus of $v_{b}$ takes into account that only the direction of $v_{a}$ matters, not the modulus. The triple integral with respect to $r_{a}$ is necesary in order to sum over all possible initial positions of the particle in the source. However, the cross-section should be independent of the density $\rho \left(r_{a}\right)$ of particles. Then, I shall assume that the velocity is in the direction of the Z axis and that the density ρ is homogeneous in a slab with limits α≤z≤β. Then, the following should be substituted for Equation (37):

(38) $$\sigma \left(\theta ,\phi \right)\propto \int_{-\infty }^{\infty }dx_{a}\int_{-\infty }^{\infty }dy_{a}\int v_{b}^{2}dv_{b}P_{v}\left(r_{a}\to v_{b}\right).$$

In order to relate $P_{v}\left(v_{b}\right)$ with the quantity $P\left(r_{a},t_{a}\to r_{b},t_{b}\right)$, Equation (35), I shall choose a coordinate system originating in (a point of) the target and ensure the initial velocity $v_{a}$ is in the positive *Z* direction, as said above. Also, I fix t=0 to be the time where the particle crosses the surface z=0 of the target, but the components *x* and *y* of its position may be arbitrary. We assume that the distances’ source-target, $L_{st},$ and target-detector, $L_{td},$ are very large in comparison with the size of the target. We shall consider only particles that cross the target region because the experimental set-up is so prepared. That is, I exclude the possibility that there are particles going directly from the source to the detector without crossing the target.

The motion of any particle will be uniform, i.e., a straight line with constant velocity $v_{a}$, from the source to a point near the target (position $r_{0})$ and also uniform from another point near the target (position $r_{n})$ to the detector with velocity $v_{b}$, because in both cases, the particle moves in a region without potential (see Equation (24)). Thus, the non-trivial part of the path of a particle, which I define by the points $\left\{r_{1},r_{2},...r_{n-1}\right\},$ takes place within the target region. I shall start from $P(r_{a},t_{a}\to r_{b},t_{b})$, Equation (35), defining

$$r_{0}=t_{a}v_{a},r_{n}=t_{b}v_{b},$$

whence we get

(39) $$P_{v}\equiv P\left(r_{a},t_{a}\to v_{b},t_{b}\right)=\varepsilon^{-3n}\int dr_{n-1}\cdots \int dr_{1}\times \prod_{j=1}^{n-1}Q_{j}.$$

That is, $P_{v}$ gives the probability that a particle initially placed in $r_{a}$ reaches the velocity $v_{b}$ after crossing the target.

Performing the integrals in $dr_{j}$ would require the form of the potential $V\left(r\right)$ in the target and would be lengthy. In fact, $Q_{j}$ is not only acting as a function of $V\left(r_{j}\right)$ but also impacting on variables like $s_{j}$ depending on $r_{j},r_{j-1}$ and $r_{j+1}$ (see Equations (35) and (36)). As a consequence, calculating $P_{v}\left(v_{b}\right)$ is complicated. In the next section, I shall use the relatively simple Born approximation, which is valid for weak potentials.

### 4.4. Born Approximation

The Born approximation for scattering is a useful application of non-relativistic quantum mechanics. In the following, I will re-derive Born’s formula from the formalism proposed above. Obviously, the aim is not to make a new derivation, but to illustrate the formalism. Born’s formula corresponds to an approximation valid for a potential that is weak and localized. For notational simplicity, in the following, I shall omit the dimensional parameters, taking m=ħ=1. These parameters will be restored at the end.

In a weak potential, it is convenient to start from Equation (35), writing

Qj=δ3sj−εFj,Fj=ImV˜2sjexp−2isj·rj.

Then, I will expand $\prod_{j=1}^{n-1}Q_{j}$ in powers of ε/2, and Born approximation consists of truncating the expansion at the minimal order of ε, which leads to a non-trivial result. It may be realized that the zeroth and first-order terms do not contribute to the cross-section. Then, I shall work with the term of second order, that is,

(40) Pv2∝∫drn−1⋯∫dr1∏k=1j−1δ3sk×εImV˜2sjexp−2isj·rj×∏p=j+1l−1δ3sp×εImV˜2slexp−2isl·rl×∏q=l+1n−1δ3sq.

As noted above, I will ignore the normalization in the following, thus substituting proportionality, ∝, for equality.

Taking the definition of $s_{k}$ Equation (23) into account, the Dirac delta $\delta^{3}\left(s_{k}\right)$ implies that the velocity does not change at any of the times $t_{k}$. Therefore, Equation (40) means that the motion of the particle is uniform until time $t_{j}$, with constant initial velocity $v_{a}$. It is also uniform from $t_{j}\equiv t^{\prime }$ to $t_{l}$ with some velocity v, and from $t_{j}$ on with the final velocity $v_{b}$. Thus, the path of the particle is defined by the points $\left\{r_{0},r_{j},r_{l},r_{n}\right\}$ with uniform motion between every two successive points. The parameters needed in order to define the path are the position vectors $r_{a},r_{j}$ and $r_{l}$ and the times $\varepsilon j\equiv t^{\prime }$ and t≡(l−j)ε. The following relations exist amongst the parameters:

(41) $$r_{a}-r_{0}=v_{a}T,r_{j}-r_{0}=v_{a}t,r_{l}-r_{j}=vt,$$

where T is the time travel from the source to the target. Then, after integrating with respect to all vector variables $\left\{r_{k},r_{p},r_{q}\right\},$ Equation (40) leads to

(42) Pvra→vb∝∫drj∫drlIm−V˜2v−2vaexp−i2v−2va·rj×Im−V˜2vb−2vexp−i2vb−2v·rl.

The integrals over $dr_{j}$ and $dr_{l}$ should be extended to the whole 3D space. I point out that the integrals in $x_{a},y_{a}$ of Equation (38) are now not needed because they were already performed in the $r_{j}$ integration. In fact, $x_{a}=$$x_{0},y_{a}$ = $y_{0}$ as a consequence of Equation (41) and the fact that $v_{a}=\left(0,0,v_{a}\right).$

In order to proceed, it is convenient to use the following expression for a product of two imaginary quantities, that is, where $a_{j}$ is complex and $b_{j}$ is real:

Ima1expib1)Ima2expib2)=12Rea1a2∗expib1−b2−12Rea1a2expib1+b2.

If we apply this equality to Equation (42), two terms appear, which I will label $P_{21}$ and $P_{22}$. The second does not contribute to the cross-section, as will be shown later. The first one may be written as

(43) P21=∫drj∫drlReJ,J≡V˜∗2vb−2vV˜2v−2vaexp2irj·v−va×exp2irl·v−vb.

I shall change the variables as follows:

(44) $$r_{+}=\frac{1}{2}\left(r_{l}+r_{j}\right),r_{-}=r_{l}-r_{j}\Rightarrow r_{l}=r_{+}+\frac{1}{2}r_{-},r_{j}=r_{+}-\frac{1}{2}r_{-},$$

where the Jacobian is unity. Then, Equation (43) becomes

P21=Re∫dr−∫dr+V˜∗2vb−2vV˜2v−2va×expir−·va−vb×exp2ir+2v−va−vb.

The integral with respect to $r_{+}$ gives

P21=4π3Re∫dr−V˜∗2vb−2vV˜2v−2va×expir−·va−vbδ32v−va−vb=4π3V˜vb−va2Re∫dr−expir−·va−vbδ32v−va−vb.

Hence, taking Equations (41) and (44) into account, we get

P21∝V˜vb−va2Re∫dr−expir−·va−vbδ32r−/t−va−vb.

The Dirac $\delta^{3}$ implies that two components of $r_{-}$ are nil, whence the triple integral reduces to a single one for the component of $r_{-}$ in the direction of $v_{a}+v_{b}.$ Then, taking the Dirac δ into account, we may substitute $\left(v_{a}+v_{b}\right)t/2$ for $r_{-}$, leading to

(45) P21∝V˜vb−va2Reva+vb/2∫dtexpiva+vb·va−vb∝V˜vb−va2δva2−vb2.

Taking Equation (38) into account, the cross-section is

(46) $$\sigma \left(\theta ,\phi \right)\propto {\left|\tilde{V}\left(v_{b}-v_{a}\right)\right|}^{2}={\left|\int dx\exp\left[-im\hbar^{-1}x\cdot \left(v_{b}-v_{a}\right)\right]V\left(x\right)\right|}^{2},$$

where the dimensional parameters have been restored. This is Born’s formula. I stress that, in our calculation, the conservation of energy, that is, $\left|v_{b}\right|=\left|v_{a}\right|,$ appears explicitly in Equation (45). For this reason, the integral $\int v_{b}^{2}dv_{b}$ of Equation (38) is irrelevant.

In texts of quantum mechanics, the Born cross-section is usually derived from the Schrödinger equation, and the result is given in terms of the wavevectors associated with the particle, that is,

$$\sigma \left(\theta ,\phi \right)=\frac{1}{16\pi^{2}}{\left|\int dx\exp\left[-ix\cdot \left(k_{b}-k_{a}\right)\right]V\left(x\right)\right|}^{2},k=\frac{mv}{\hbar }.$$

See [7].

The calculation of the term $P_{22}\left(v_{b}\right)$ is similar to the one of $P_{21}\left(v_{b}\right)$ Equation (43), but I will skip the details. The main difference is that $\delta^{3}\left(v_{b}-v_{a}\right)$ is substituted for $\delta^{3}\left(2v-v_{a}-v_{b}\right).$ This implies that the velocity does not change in the scattering process, and therefore, the term $P_{22}\left(v_{b}\right)$ does not contribute to the desired cross-section.

### 4.5. Interference Experiment

Born’s approximation allows us to calculate the result of simple interference experiments. For instance, let us consider a particle with initial velocity $v_{0}=\left(0,0,v_{0}\right)$ that is moving in the *Z* direction and arrives at a region with the potential

(47) $$V\left(r\right)=C\left\{\exp\left[-\lambda {\left(r+a\right)}^{2}\right]+\exp\left[-\lambda {\left(r-a\right)}^{2}\right]\right\},a\equiv \left(a,0,0\right),$$

which is a model for a screen with two holes, appropriate for a simple calculation. Obtaining the cross-section via Born’s approximation is not difficult using Equation (46). We get

$$\sigma \propto \exp\left[-\frac{v^{2}+v_{0}^{2}-2v_{0}v_{z}}{2\lambda }\right]\cos^{2}\left(av_{x}\right),$$

where $v=\left(v_{x},v_{y},v_{z}\right)$ is the final velocity. Hence, taking the conservation of energy, Equation (45), into account, the cross-section becomes

(48) $$\sigma =C^{2}\frac{\pi }{\lambda^{3}}\exp\left[-\frac{2v_{0}^{2}\sin^{2}\theta }{\lambda }\right]\cos^{2}\left(av_{0}\sin\theta \cos\phi \right).$$

Assuming that the particles detected consist of spots produced in a screen parallel to the XY plane, we would observe typical interference fringes with a decreasing intensity in both directions, *X* and *Y*, with a maximum at x=y=0.

The point of this calculation is that no waves are involved, only particles. Therefore, in our approach, the wave behaviour in the interference is an effect of the non-local action of the potential. Of course, we might assume that the action is mediated by some “hidden” waves that do interfere, which fits in with the assumption that quantum particles are not simple objects, as is the case in the classical view. They are dressed by quantum fields that give stochastic and non-local features to the motion [8,9].

### 4.6. Discussion

I have shown that in, non-relativistic quantum mechanics (without spin), it is possible to picture the transition probability in terms of particle paths. A path may be defined by the positions $\left\{r_{a}\equiv r_{0},\ldots r_{j},\ldots r_{b}\equiv r_{n}\right\}$ at times $t_{a,}\ldots t_{j}\equiv t_{a}+j\varepsilon ,\ldots t_{b}$ or, what is equivalent, the initial and final positions plus the velocity changes $\left\{s_{j}/\varepsilon \right\}$ at times $t_{j}.$ Eventually, we should consider the limit n→∞ with $n\varepsilon =t_{b}-t_{a}.$ This might provide a stochastic (non-local) picture where particles travel along continuous paths.

In summary, the formalism suggests an intuitive picture of non-relativistic quantum mechanics of particles in terms of the probabilities of the possible paths of *particles*. The particle’s motion is represented by a stochastic process such that there is a random change of velocity at every time $t_{j}$, with a probability depending on *the potential over a large region* around the position of the particle (indeed, it derives from the Fourier transform of the potential; see Equation (35)). The wave behaviour, e.g., in experiments of atom interference, may be interpreted assuming that the motion of the particles is governed by a law (different from Newton’s) where the “acceleration” depends on the potential on a whole spatial region, at odds with the local action of classical dynamics. With this interpretation, the interference experiments with particles (e.g., atoms) might be explained without assuming that those particles possess a wave nature or that they may cross two distant slits at the same time. But I stress again that we remain at the level of non-relativistic quantum mechanics. I do not claim that a similar interpretation may be extended to relativistic quantum field theory when spin plays a role, or even to atoms or molecules when (Bose or Fermi) statistics is relevant. We have dealt with a single particle, but the generalization to *N* interacting particles is straightforward, except for the possible effects of quantum statistics.

In summary, it is not obvious whether the development of Feynman’s formalism presented herein possesses a physical interest or it is just a mathematical exercise. In any case, it illustrates the fact that the interpretation of quantum mechanics suggested by the Hilbert space formalism may not be the sole possible one.

## 5. Comments on Previous Studies of Quantum Particles Motion

Quantum mechanics does not describe the evolution of physical systems in terms of particles, at odds with classical mechanics. Indeed, in the abstract, Hilbert space formalism encompasses only the states of the system and the observables that appear, which are represented by vectors and operators respectively. In the Schrödinger representation, the states look like waves, represented by wavefunctions, and the observables become differential operators. The particle aspect appears only via Born’s rule, which relates the wavefunction with a probability distribution for the positions of particles. However, our intuition and the classical explanation of many phenomena strongly suggest that particles are real. In particular, they suggest that ordinary matter consists of atoms and molecules. This fact has lead people to attempt to describe quantum phenomena in terms of the motion of particles, provided that the results obtained agree with the quantum predictions. I may quote at least four different proposals for quantum particle motion: Bohmian mechanics, stochastic mechanics, the Wigner representation, and the so-called decoherent consistent trajectories.

Bohm [10,11] started from the Schrödinger wavefuction written in polar form, which is

$$\psi \left(r,t\right)=\sqrt{\rho \left(r,t\right)}\exp\left[iS\left(r,t\right)/\hbar \right],$$

and proposed that it represents the motion of a quantum particle along different possible paths. In every path, the velocity of the particle placed at $\left(r,t\right)$ is given by

(49) $$v\equiv \frac{dr}{dt}=-\frac{1}{m}▽S\left(r,t\right).$$

The result is a theory that provides more information than quantum mechanics but agrees with QM in all predictions of the latter. Thus, it is a typical instance of a “hidden-variable theory” (HVT); indeed, it is the paradigmatic example. HVTs were proposed in the early period of QM, and they have a long history (see, e.g., a recent Oxford Handbook on interpretations of QM [12]).

Bohmian mechanics presents several shortcomings. For instance, in stationary states, the S phase of the wavefunction does not depend on the coordinates; hence, the Bohmian particle is at rest, but the theory does not provide clues about the reason for the stationary probability distribution $\rho \left(r\right).$ Also, solving Equation (49) requires the previous solution of Schrödinger’s equation. At odds with the approach of this paper, the probabilities of paths may be calculated from the potential $V\left(r\right).$

Stochastic mechanics rest on the assumption that the motion of a quantum particle may be treated as a stochastic process [13,14]. The theory had some successes, like the derivation of the Schrödinger equation, but also difficulties [14]. It has some similarity with Bohmian mechanics, with the advantage being that the paths present stochasticity, which fits the probabilistic character of QM. Obtaining an intuitive interpretation of the physics in Nelson’s theory is less obvious than in our approach.

The Wigner representation rests on the transform proposed by Weyl [15] and Wigner [16] that relates both states and observables with functions in the phase space of coordinates and the momenta of the particles. The evolution is obtained from an appropriate transform of the Schrödinger equation, whence it is possible to derive the motion of the quantum particles. The Wigner representation provides a formulation of QM equivalent to the standard one [17], but it presents calculational advantages only in some particular cases. On the other hand, it does not provide an intuitive interpretation of QM because the phase space functions representing states are not positive in general, so they cannot be interpreted as probability distributions. In sharp contrast, the Wigner representation of the electromagnetic field provides a realistic interpretation of the quantum field [8,9].

In summary, I believe that the formalism developped in this paper for the motion of quantum particles is superior to the three quoted approaches. However, the Wigner representation is interesting for other reasons, as mentioned above.

Consistent decoherent history interpretation of quantum mechanics does not really deal with the motion of quantum particles. Decoherence has been introduced in the study of quantum entanglement for the macroscopic or mesoscopic domain. There, paradoxes arise, as exhibited by the celebrated Schrödinger’s cat problem, in which, according to QM, the cat may exist in a coherent superposition of alive and dead. The solution appeals to the relevance of the environment, unavoidable for macroscopic bodies, which leads to rapid decoherence [12]. The approach of consistent histories tries to study the quantum evolution, not just particle motion. The aim is to introduce probabilities into quantum mechanics in a fully consistent and physically meaningful way and ensure that they can be applied to a closed system, dispensing the notion of measurement and consequently dissolving its inevitable paradoxes. See, e.g., the paper authored by Rocha et al. [18] and the references therein.

## Data Availability

No new data were created or analyzed in this study. Data sharing is not applicable to this article.
